# Quantifying the Diagnostic Odyssey Burden Among Persons with Inborn Errors of Immunity

**DOI:** 10.1007/s10875-024-01855-x

**Published:** 2025-01-02

**Authors:** Sarina Nikzad, Rebekah Johnson, Christopher Scalchunes, Nicholas L. Rider

**Affiliations:** 1 Liberty University College of Osteopathic Medicine, Lynchburg, Virginia USA; 2 Immune Deficiency Foundation, Hanover, Maryland USA; 3 Department of Health Systems & Implementation Science, Virginia Tech Carilion School of Medicine, Roanoke, Virginia USA; 4 Section of Allergy-Immunology, Department of Medicine, The Carilion Clinic, Roanoke, Virginia USA

**Keywords:** Inborn errors of immunity, Primary immune disorders, Patient reported health, Diagnostic odyssey, Health burden

## Abstract

**Purpose:**

Patients with inborn errors of immunity (IEI) have lifelong health complications including severe infections and physical impairments. Previous studies show that a patient’s perception of their health is an important predictor of health outcomes. The purpose of this study was to understand factors related to patient reported health status.

**Methods:**

We used data from the Immune Deficiency Foundation (IDF) 2017 National Patient Survey and analyzed factors which correlated with the reported health status (RHS). Among a cohort of 1139 self-reported IEI patients, we identified age at the time of diagnosis, time gap between symptom onset and diagnosis, number of physicians seen, and whether the diagnosis was made in the first 5 years of life as significant. We used a two-tailed t-test, single-factor ANOVA, and Tukey-Kramer post-hoc test to assess statistical significance in the observed difference.

**Results:**

Patients who received a diagnosis before the age of 12 had a significantly better mean RHS (*n* = 207 pre-12a vs. *n* = 900 post-12a; *p* < 0.0001). Patients who received a diagnosis within 10 years of symptom onset showed improved mean RHS (*n* = 413 pre-10 vs. *n* = 524 post-10; *p* < 0.0001). Among patients who had symptom onset within the first 5 years of life, those who received a diagnosis had a significantly improved RHS (3.5 ± 0.92, *n* = 275 undiagnosed vs. 2.8 ± 0.94, *n* = 108 diagnosed; *p* < 0.0001). Finally, RHS was significantly impacted by number of physicians(*n* ≥ 4) seen prior to diagnosis (3.1 ± 0.96 vs. 3.4 ± 0.80, *p* < 0.0001).

**Conclusion:**

These findings shed light upon critical factors which impact IEI patient RHS. Specifically, we find that efficient, rapid and early-life IEI identification should improve patient reported health and relevant outcomes. These improvements appear to be independent of the clinician specialty ultimately making the IEI diagnosis.

**Supplementary Information:**

The online version contains supplementary material available at 10.1007/s10875-024-01855-x.

## Introduction

Inborn errors of immunity (IEI) are a group of chronic conditions characterized by a single gene mutation leading to a defective immune system of the affected individuals [1]. Although individually rare, as a group, IEI affects approximately 200,000 people across the United States [2]. Patients suffer from an extensive list of health conditions including an increased susceptibility to infections, autoimmune diseases, and malignancies [1]. However, each patient’s journey is unique, and surveys have shown a wide variance in how patients perceive their overall health [3]. Looking at perceived health allows us to analyze the importance of different variables and their distinct impact on health outcomes for IEI patients.

Perceived health (PH) is a subjective measure of individuals’ overall wellbeing and studies have shown it to be a reliable indicator of many objective health outcomes including morbidity and mortality [4]. Similarly, it has been observed that patients’ in better health have reported a higher satisfaction with their health status [4, 5]. Many factors have been shown to affect PH including education level, hospitalization, and access to specialist care [3]. When caring for patients with chronic conditions such as IEI, understanding all that affects their physical, mental, and overall health status allows providers to deliver a more personalized and more effective care to their patients.

Patients can communicate their PH through regular health surveys, which is common practice among Organization for Economic Co-operation and Development (OECD) countries. A standard question for assessing PH is “How is your health in general?” and patients can choose between Excellent, Very Good, Good, Fair, or Poor. This subjective reported health status (RHS) is correlated with healthcare utilization and mortality. Several studies have explored the relationship between RHS and variables such as socioeconomic, psychosocial, behavioral factors [6–8].

In this study we aimed to explore other factors that potentially affect the RHS of patients with IEI. These factors include age at the time of diagnosis, time gap between symptom onset and receiving a diagnosis (i.e. “diagnostic gap”), having a diagnosis in the first 5 years of life, the number of physicians seen, and the type of specialist that provided the diagnosis. A priori, these factors are expected contributors to impaired RHS; however, the degree of impact has not been evaluated. Previous studies on RHS in patients with IEI showed that earlier detection is vital and leads to less morbidity. These studies also stressed the importance of “vigilant care” which prevents further complications and serious healthcare interactions in the future [9]. Knowing important variables and understanding how they impact patients with IEI is vital for both quality patient care and health outcomes. Many studies reinforce this importance for patients with IEI [9–11].

## Methods

Data for this study was collected from the Immune Deficiency Foundation (IDF) 2017 National Patient Survey Data Dictionary. The survey coded the health status (RHS) of the patients on a 5-point scale, 1 for excellent health and 5 for poor health. We conducted analyses which addressed hypotheses related to patient age at diagnosis, diagnostic gap, impact of serious infections at the time of diagnosis and diagnostic odyssey burden impact upon patient reported RHS.

### Age-Based Cohort Comparisons

We stratified the patients based on their age at the time of diagnosis into 6 groups: 0–12, 13–30, 31–45, 46–55, 56–65, > 65 years of age. Age groups were selected to ensure a similar number of subjects in each group. The mean RHS of the groups were compared using a single factor ANOVA. Pairwise group comparison was then conducted via a Tukey-Kramer post-hoc test to identify group by group differences.

### Calculating the Diagnostic Delay Timespan(“Diagnostic Gap”)

To determine the diagnostic gap, we indexed age of first serious infection (indicator of symptom onset), age of IEI diagnosis and calculated the difference. We then split the patients into 5 diagnostic gap groups: <1, 1–10, 11–25, 26–40, and > 40 years elapsed. Mean RHS of the groups were compared using a single factor ANOVA. Pairwise group comparisons were then conducted via a Tukey-Kramer post-hoc test to identify group by group differences.

### Quantifying the Diagnostic Odyssey Burden

We first assessed burden by impact of serious infection and lack of diagnosis. For this analysis, the patients were stratified by age of serious infection first reported at 5-year intervals, from 0 to 90. The proportion of subjects in each age bracket with serious infections were then calculated and the temporal association between health status measurement and timing of their IEI diagnosis was determined. From this, we focused our analysis on the 0–5-year age group, which had the largest number of patients experiencing serious infections (*n* = 383). However, within that group of 383 patients experiencing serious infections there were only 108 who had received a diagnosis. We then compared the group mean RHS of patients who experienced serious infections within that range (0–5 yrs) with a diagnosis (“Dx”; *n* = 108) to those without (“UDx”; *n* = 275).

Next, we evaluated burden by the number of physicians seen. As a group(*n* = 1145), the mean number of physicians seen per patient was calculated (*n* = 3) and the patients were divided into groups who had seen $\:\le\:\:$3 physicians and those who were seen by 4 or more physicians. A Student’s t-test was then used to compare mean RHS of patients across the two groups.

## Results

Demographics of patients studied in this cohort is described in Table 1. Out of 1145 patients and parent proxies, 1139 reported their health status:38 patients reported excellent health (3%), 196 reported very good health (17%), 435 reported good health (38%), 372 reported their health as fair (33%), and 98 reported poor health (9%). The average RHS of the population was 3.259 ± 0.953.

**Table 1:** Demographics of the population

Population Demographics	Total (%)
Average Age	39.55+/-21.54
Male (Self-Reported)	272 (24%)
Female (Self-Reported)	831 (73%)
American Indian/Alaskan	12 (1%)
Asian/Pacific Islander	10 (1%)
Black	6 (0.5%)
Latin	19 (2%)
White	1071 (94%)
Two or More Races	24 (2%)
Other Race	3 (0.5%)
Patients with CVID^*^	663 (48%)
Patients with Hypogammaglobulinemia	216 (15%)
Patients with IgG Subclass Deficiency	137 (10%)
Patients with Other IEIs^**^	364 (26%)
Total number of patients studied	1139

*CVID = common variable immunodeficiency

**Other IEIs described on the surveys include: agammaglobulinemia, ataxia telangiectasia, chronic granulomatous disease, combined immunodeficiency, complement deficiency, DiGeorge anomaly, hereditary angioedema, hyper IgE syndrome, hyper IgM syndrome, selective IgA deficiency, severe combined immunodeficiency, severe congenital neutropenia, specific antibody deficiency, Wiskott-Aldrich syndrome, other IEI, and unspecified diagnoses. See supplemental materials for further details

### Age-Based Cohort Comparisons

A total of 1107 patients reported age at diagnosis and were included in this analysis. The primary analysis (e.g. ANOVA) showed a significant difference between mean RHS across all age groups (*p* < 0.0001). Pairwise post testing revealed that significance stemmed from the 0–12 group which had a significantly better RHS compared to other groups individually (Table 2).

**Table 2:** Mean reported health status at age of diagnosis

Age Groups (Years)	Count (*n*)	Mean RHS	95% Confidence interval	ANOVA *p*-value
0–12	207	2.7^*^	0.84–4.56	*p* < 0.0001
13–30	117	3.5	1.28–5.25	
31–45	259	3.3	1.61–5.37	
46–55	229	3.3	1.82–5.16	
56–65	212	3.5	1.45–5.15	
> 65	83	3.2	1.63–4.83	
Overall	1107	3.2	1.43–5.06	

*Mean RHS that was significantly different from the others by Tukey-Kramer post-hoc test.

### Calculating the Diagnostic Delay Timespan(“Diagnostic Gap”)

Of the 1107 patients who met the criteria for the first analysis, 937 reported the age of symptom onset and were studied in this analysis. There was a significant difference between the RHS across all groups (*p* < 0.0001). Group pairwise comparison demonstrated significant RHS differences between patients with a diagnostic gap $\:\le\:10$ years (i.e. groups <1 and 1–10 yrs) compared to those with the longest diagnostic gap (i.e.>40 years elapsed) (Table 3).

**Table 3:** Reported Health Status and the diagnostic gap

Diagnostic Gap (Years Elapsed)	Count (*n*)	Mean RHS	95% Confidence interval	ANOVA *p*-value
< 1	78	3.1^*^	0.87–5.23	*p* < 0.0001
1–10	335	3.1^*^	1.16–5.10	
11–25	203	3.4	1.65–5.06	
26–40	168	3.2	1.42–5.06	
> 40	153	3.5	1.53–5.40	
Overall	937	3.3	1.32–5.28	

*Mean RHS that was significantly different from the others by Tukey-Kramer post-hoc test

### Quantifying the Diagnostic Odyssey Burden

Of 383 patients who experienced serious infections in the 0–5 age range, 108 (28%) had received an IEI diagnosis while 275 (72%) had not. Between these two groups, we found a significant difference in reported health scores when comparing diagnosed (Dx) and undiagnosed (UDx) patients (Table 4). Here, we note that the 0–5 year-olds with serious infections having received a diagnosis had a significantly better RHS score than those who went undiagnosed.

Among the entire cohort (*n* = 1145) the mean and median number of physicians seen was 3(mean: 3±2.4; range: 0–11). Mean RHS among those who saw 3 (*n* = 617) or fewer physicians vs. those that saw 4 (*n* = 521) or more was significant (3.1 ± 0.96 vs. 3.4 ± 0.80, *p* < 0.0001). Here, RHS scores were improved among those seeing fewer physicians.

**Table 4:** Reported Health Status & Importance of receiving a diagnosis

Patient category	Count	Mean RHS	t-test *p* value
Undiagnosed	275	3.5 ± 0.92	*p* < 0.0001
Diagnosed	108	2.8 ± 0.94	

## Discussion

This analysis used data derived from a nationally distributed mail/postal survey conducted by the IDF which captures a variety of first-person perspectives from IEI patients about their own health [12]. These surveys inquire about many different aspects of patients’ lives including their quality of life and how they perceive their general health. In our study, RHS was determined based on an answer to the question “ In general, how would you say the patient’s current health is?”. This question has been described as a reliable measure of health status as it pertains to both the objective wellbeing and subjective perceived health [12, 13]. Studies have shown a relationship between RHS and patient outcome and morbidity, supporting that a better RHS is indicative of better health [13, 14]. In this study we analyze and demonstrate, for the first time, the relationship between RHS and factors associated with a patients’ journey to receiving a diagnosis.

From our analysis, we wish to emphasize several key findings, First, time to diagnosis matters for IEI patients. Our analysis reveals that having a diagnosis sooner, and at a younger age led to better RHS scores further emphasizing a need for optimizing early IEI detection(Table 2). Even a diagnostic gap of ten years or less had significantly improved RHS compared to those diagnosed over longer intervals of time(Table 3). While a diagnostic gap of 10 years is still an interminable amount of time, we note the quantitative impacts of longer durations upon RHS here. In terms of age at diagnosis, patients with the lowest (i.e. better) RHS came from the youngest age bracket (Table 2; age 0-12yrs). Additionally, many patients in this cohort started experiencing infections at a young age which probably improved their likelihood of receiving an IEI diagnosis. However, within the 0–5 age group(*n* = 383), less than a third (*n* = 108; 28%) received an IEI diagnosis(Table 4). Among those, we found a significantly better RHS compared to those in the same age group without a diagnosis. These findings suggest that receiving a diagnosis at an earlier age and through a shorter diagnostic gap is associated with lower (i.e. better) RHS scores. Quantitatively, this analysis further underscores the association between worse RHS and suboptimal patient health outcomes such as being older at diagnosis, having greater comorbidities and taking more time to receive a diagnosis [5, 13].

Finally, patients in the IDF data set saw up to 11 different specialists throughout their diagnostic odyssey. Our analysis found that seeing fewer physicians correlated with an improved RHS score. Unsurprisingly, these findings suggest that reported health diminishes, and burden increases, as patients see more and more healthcare providers along their diagnostic journey. While economic factors were not assessed here, we also expect that worse RHS and higher health expenditures would go hand in hand, further diminishing the value of care. Interestingly, we did not note a significant difference in RHS scores between those diagnosed by an immunologist compared to other physician specialties. These findings suggest that the burden came from the number of physicians seen and not necessarily any one specialty. Thus, it may not matter who makes the diagnosis so long as one is precisely made at the earliest age, with less time in the healthcare system. This notion supports efforts to build awareness across any specialty, including primary care, for improving IEI patient reported health.

From our analysis, we find that each IEI patient has a unique diagnostic journey encompassing numerous factors such as lingering infections, seeing many specialists, and suffering extended periods of time without a diagnosis. Do these factors contribute to their overall health outcome? This study suggests that RHS scores are indeed influenced by discrete features of the clinical journey. Understanding these, and other, precise factors affecting health outcomes for patients with IEI could enable efforts to address drivers of impaired RHS. Systematically, decreasing the time to treatment, reducing the number of physicians seen prior to diagnosis, and identifying IEI earlier in life are all valuable for patients. Thus, efforts aimed at mitigating these drivers of burden are expected to improve RHS and lead to better health outcomes for patients.

While we believe that our findings quantitatively bolster previously held notions about diagnostic burden, we acknowledge some limitations of this study. First, our sample may not be representative of the general population given this largely white, female cohort suffering predominantly antibody deficiency syndromes (Table 1). Second, the IDF phone survey lends itself towards selection bias, recall bias, and other forms of response bias. Additionally, we chose parametric statistical tests to analyze ordinal Likert scores because of the size and largely normal distributions within our datasets. For these reasons, our results should be interpreted as exploratory rather than confirmatory. Finally, the 2017 survey was conducted before the COVID-19 pandemic; thus, impacts of a global pandemic could drastically change burden and result in different responses not captured in this analysis.

Ultimately, we believe that findings described here show a significant need for comprehensively educating generalists and subspecialists alike to expedite IEI patient diagnosis early in life and prior to onset of morbidity. Strategies like newborn screening and computational screening (e.g. artificial intelligence based methods) in addition to focused educational initiatives, are expected to impact this goal and improve RHS concomitantly with improved outcomes [15–17].

## Electronic Supplementary Material

Below is the link to the electronic supplementary material.

Supplementary Material 1

## Data Availability

This is an observational study. Data from the IDF are available upon request.
