# Correction to: Quantifying the Diagnostic Odyssey Burden Among Persons with Inborn Errors of Immunity

**DOI:** 10.1007/s10875-025-01859-1

**Published:** 2025-01-29

**Authors:** Sarina Nikzad, Rebekah Johnson, Christopher Scalchunes, Nicholas L. Rider

**Affiliations:** 1 Liberty University College of Osteopathic Medicine, Lynchburg, VA USA; 2 Immune Deficiency Foundation, Hanover, MD USA; 3 Department of Health Systems & Implementation Science, Virginia Tech Carilion School of Medicine, Roanoke, VA USA; 4 Section of Allergy-Immunology, Department of Medicine, The Carilion Clinic, Roanoke, VA USA

**Correction to: J Clin Immunol2025 Jan 2;45(1):61.**

10.1007/s10875-024-01855-x

In this article the organization “Immune Deficiency Foundation (IDF)” was incorrectly named.

[Current verbiage Error in Abstract]

**Methods**

We used data from the Immunodeficiency Foundation (IDF) 2017 National Patient Survey and analyzed factors which correlated with the reported health status (RHS). Among a cohort of 1139 self-reported IEI patients, we identified age at the time of diagnosis, time gap between symptom onset and diagnosis, number of physicians seen, and whether the diagnosis was made in the first 5 years of life as significant. We used a two-tailed t-test, single-factor ANOVA, and Tukey-Kramer post-hoc test to assess statistical significance in the observed difference.

[Corrected verbiage in Abstract]

**Methods**

We used data from the Immune Deficiency Foundation (IDF) 2017 National Patient Survey and analyzed factors which correlated with the reported health status (RHS). Among a cohort of 1139 self-reported IEI patients, we identified age at the time of diagnosis, time gap between symptom onset and diagnosis, number of physicians seen, and whether the diagnosis was made in the first 5 years of life as significant. We used a two-tailed t-test, single-factor ANOVA, and Tukey-Kramer post-hoc test to assess statistical significance in the observed difference.

[Current verbiage Error in Methods]

**Methods**

Data for this study was collected from the Immunodeficiency Foundation (IDF) 2017 National Patient Survey Data Dictionary. The survey coded the health status (RHS) of the patients on a 5-point scale, 1 for excellent health and 5 for poor health. We conducted analyses which addressed hypotheses related to patient age at diagnosis, diagnostic gap, impact of serious infections at the time of diagnosis and diagnostic odyssey burden impact upon patient reported RHS.

[Corrected verbiage in Methods]

**Methods**

Data for this study was collected from the Immune Deficiency Foundation (IDF) 2017 National Patient Survey Data Dictionary. The survey coded the health status (RHS) of the patients on a 5-point scale, 1 for excellent health and 5 for poor health. We conducted analyses which addressed hypotheses related to patient age at diagnosis, diagnostic gap, impact of serious infections at the time of diagnosis and diagnostic odyssey burden impact upon patient reported RHS.

The original version has been corrected.

