# Supplementary material for: Quantifying the Diagnostic Odyssey Burden Among Persons with Inborn Errors of Immunity
Source: J Clin Immunol. 2025 Jan 2;45(1):61. doi: 10.1007/s10875-024-01855-x (PMC11695393; doi:10.1007/s10875-024-01855-x)
Supplement: Supplementary file 1 — Supplementary Material 1 [file 10875_2024_1855_MOESM1_ESM.docx]

| **Patients with other IEIs** | **Number of individuals** |
| --- | --- |
| **Agammaglobulinemia** | 43 |
| **Ataxia Telangiectasia** | 1 |
| **Chronic Granulomatous Disease** | 17 |
| **Combined Immunodeficiency** | 13 |
| **Complement Deficiency** | 12 |
| **DiGeorge Anomaly** | 1 |
| **Hereditary Angioedema** | 3 |
| **Hyper IgE Syndrome** | 9 |
| **Hyper IgM Syndrome** | 10 |
| **Selective IgA Deficiency** | 91 |
| **Severe Combined Immunodeficiency** | 24 |
| **Severe Congenital Neutropenia** | 2 |
| **Specific Antibody Deficiency** | 77 |
| **Wiskott-Aldrich Syndrome** | 4 |
| **Unspecified/unknown** | 57 |
| **Total** | 364 |
